# A report on the impact of CGF on hard and soft tissues around endo-perio lesion

**DOI:** 10.6026/973206300200483

**Published:** 2024-05-31

**Authors:** Sai Krishna Bingi, K Sitamahalakshmi, M Jaya Nagendra Krishna, Shaik Imran, Govardhani Krishnakumar

**Affiliations:** 1 Department of Conservative Dentistry & Endodontics, Kamineni Institute of Dental Sciences, Narketpally, Nalgonda, India; 2 Department of Periodontology & Implantology, Kamineni Institute of Dental Sciences, Narketpally, Nalgonda, India; 3 Department of Periodontology & Implantology, Government Dental College and Hospital, Vijayawada, Andhra Pradesh, India

**Keywords:** Concentrated growth factor (CGF), endo perio lesion, regeneration, bone volume, platelet concentrates generation

## Abstract

There is an increased chance of further periodontal deterioration due to severe intrabony defect. There are s different patho physiologies for perio-endo lesions, ranging from quite basic to rather complicated but to make the right diagnosis, one must be aware of various illness processes also a careful history taking, examination, and the application of specialized tests can help achieve this. Each form of endodontic-periodontal illness has a different prognosis and course of therapy and all kinds of endo-perio lesions require endodontic and periodontal treatments are necessary for primary periodontal disease with subsequent endodontic involvement and real mixed endodontic-periodontal disorders. The severity of the periodontal disease and how well the patient responds to therapy will determine how these situations turn out. Because autologous platelet concentrates are enriched with growth factors, such as concentrated growth factor (CGF), they may enhance surgical outcomes. CGF is inserted into the appropriate intrabony defect following traditional flap debridement. Following flap surgery, the tooth in question had a root canal operation. Volumetric analysis was performed on both groups before to surgery and nine months after the procedure. It has been discovered that the defect area has a much larger bone volume due to the high levels of CGF, a regenerative and reconstructive growth factor that promotes early and high bone fill.

## Background:

The cellular complexes that make up the pulp and periodontal tissue are closely connected and distinct. There are several routes for physiologic communication, including the apical foramen, lateral and auxiliary canals, and exposed dentinal tubules [1]. Endodontic-periodontal lesions (EPLs) are those tissues that are affected in both systems, and they can occur alone or in combination [2]. The EPL has consistently presented a clinical challenge for many years. It depicts a pathologic route that can be triggered by many etiologies that connects the pulp of a tooth to the periodontal tissue. Caries, trauma, restorative operations, chemicals, or extreme heat stimulation that affects the pulp and, secondarily, the periodontium can all lead to the EPL [3]. A second etiology of endodontic problem might be periodontal disease, which mostly affects the root canals. This periodontal lesion is caused by calculus and plaque. The exposed dentinal tubules on the cementum's missing sections will be more vulnerable to bacterial invasion, which will raise the possibility of the pulp suffering cumulative damage that would result in retrograde pulpitis. Pulpal necrosis can result from periodontal disease that advances apically and affects the apical foramen [4]. Correct identification of the etiology of EPL is necessary for an effective treatment approach. Clinicians ought to be well-versed on this lesion scientifically and intuitively, regardless of their specialty [5]. Since ancient times, periodontal flap surgery has been used to manage bone loss. To improve the bone at the defect site, a variety of materials have been employed. Platelet concentrates including vascular endothial growth factor(VEGF) and transforming growth factor-beta (TGF-β1) have been demonstrated to be essential for angiogenesis, neovascularization, and vascular maintenance [6]. Therefore, it is of interest to report on the impact of CGF on hard and soft tissues around endo-perio lesion.

## Materials and Methods:

One intrabony defect is assessed in a 30-year-old male patient on one site i.e:46 who are systemically healthy and no adverse habits found. Radiographically radiolucency found at 46 site involving furcation and diagnosed as perio-endo lesion.

## Parameters related to clinical practice:

## Probing Depth:

This is the line between the free gingival margin and the pocket's base. The computation included deducting (FRP-GM) from (FRP-BOP).

## Indices:

Plaque index was calculated using Silness & Loe's criteria overall mentioned in Table 1. By summing together all the scores and dividing by the total number of surfaces, the PI was determined. A plaque index of 0.1 to 0.9 is good dental hygiene; 1.0 to 1.9 is acceptable dental hygiene and 2.0 to 3.0 is bad dental hygiene. Loe and Silness gingival index is given in Table 2 Mesiobuccally, mid buccal, distobuccally, and lingual are the four scoring units that make up the tissue that surrounds each tooth. Every region was assessed clinically, questioned, and given a score depending on the severity of gingivitis is divided into three categories: mild, moderate, and severe (0.1-1.0), 1.1-2.0, and 2.1-3.0.

## Radiographic parameters:

This is measured from the cementoenamel junction (CEJ) to the base of the defect (BOD). Computing volumetric bone fill and linear bone growth is done. The measures are linear. The linear bone growth was calculated using the CEJ to BOD ratio. By deducting the baseline CEJ to BOD from the 9-month CEJ to BOD, linear bone growth was computed. Volume rendering function in CBCT In vivo software was used to compute volumetric bone fill in three dimensions.

## Treatment protocol:

Procedure:

Baseline Initial Visit (0 weeks): To evaluate the state of the periodontal tissues, PD, PI, and GI were noted. Following an evaluation of their oral hygiene maintenance, surgery was only permitted for individuals who maintained exceptional oral hygiene (PI < 1). Incisions were created in the buccal and lingual sulcular areas, and mucoperiosteal flaps were elevated. With considerable caution, the largest amount of interproximal soft tissue was retained. The defects were fully removed using ultrasonic technology and manual curettes, and root planing and scaling were used to guarantee smooth roots. The affected area had open flap debridement, and CGF was inserted and sutured with the pack. Patients were given 500 mg of Amoxicillin eight hours a day for five days after surgery, along with 50 mg of the painkiller Diclofenac sodium eight hours a day. If the individuals had discomfort afterwards, they were told to take an analgesic. Following flap surgery, the patient had root canal therapy for the affected tooth. The patient was subsequently sent to an endodontist for root canal therapy (RCT), which was carried out with the ProTaper Universal System (Dentsply Sirona). The drug UltracalTM XS Calcium Hydroxide Paste (Ultradent) was used in between visits. The tooth was obturated with AH Plus sealer (Dentsply Sirona) and a single cone obturation procedure using gutta-percha. Nine months later, the patient was brought back to analyze the parameters.

## Results and Discussion:

The purpose of the current study is to assess the efficacy of platelet concentrate production in cases with osseous anomalies or periodontal intrabony. Platelet concentrates can reduce pain, promote healing, inhibit tissue adhesion, stop bleeding, and accelerate the formation of new tissues since they are a rich source of growth factors. Utilizing the patient's own blood, platelet concentrate is a biological product that provides benefits such as reduced bleeding, decreased scarring, and serous fluid collection [7]. Thus, this study evaluated the effectiveness of CGF in correcting periodontal intrabony osseous defect following endodontic therapy. There is a difference and enhancement at 9 months when compared to baseline in PI, GI, PPD and values mentioned in Table 3. There is a higher improvement in bone volume from base line to 9 months (Table 4). A volumetric analysis of intrabony defects using CBCT was carried out for more accurate radiography readings. In this study, soft tissue was examined using PI, GI, and PPD. Parameters were assessed nine months after baseline. PI, GI, and PPD level reductions from baseline to nine months. Many growth factors, including transforming growth factor-β (TGF-β), platelet-derived growth factor (PDGF), and vascular endothelial growth factor (VEGF) [8], may contribute to the development of new tissue. The growth factors are attached to a thick network of fibrinous scaffolds, delaying their release and preventing early proteolysis. By doing this, the greatest outcomes for both immediate and long-term wound healing are guaranteed. This might also be the result of well executed endodontic surgery following periodontal surgery, which permits total infection eradication and creates an environment conducive to healing and regeneration. The endodontic component would recover with appropriate endodontic therapy, and the prognosis would ultimately rely on how well either of the therapeutic modalities handled the periodontal repair or regeneration [9]. In the current study, CBCT was utilized to do bone volumetric analysis utilizing software. The results showed that both groups' bone volume increased from baseline to nine months. Nine months after the baseline, the amount of bone has increased noticeably. This may be the case because CGF promotes osteoblast proliferation and bone repair, which speeds up Osseo integration. Angiogenesis and tissue remodeling are facilitated by CGF, which consists of fibrinogen, growth factors, leukocytes, coagulation factors, endothelial growth factors, and platelets [10]. Scarring is reduced by CGF because of its various advantages, which include increasing osteogenesis and wound healing, speeding up epithelial, endothelial, and epidermal regeneration, and possessing properties that aid in homeostasis and tissue repair. Strong antibacterial properties are conferred by its high leukocyte concentration, which also serves as a scaffold to encourage cytokines and cellular movement. Its moldable quality and strong interwoven fibrin network make it potentially useful for treating a variety of shaped bone abnormalities. By trapping platelets and leukocytes in the fibrin network to release the growth factor, it accelerates bone regeneration and does away with the need for titanium mesh or bone tack [11]. Due of its numerous advantages, which include encouraging it doesn't require any biochemical additives to produce, and its strong fibrin interaction minimizes the creation of soft tissue. The mineral scaffold contains growth factors and bone cells, both of which are necessary to produce new bone, and both encourage cells [12]. These align with the research conducted by Sitamahalakshmi *et al.* [13].

## Conclusion:

Regenerative therapy is one line of treatment that can be suggested to patients for the management of intrabony defects involving endodontically while using platelet concentrations like as CGF can be used as an adjuvant to replace collagen membrane in periodontal regeneration without compromising clinical outcomes, even if resorbable GTR is still the preferred method for regeneration. The current situation highlights how crucial it is to comprehend the nature of EPL but thorough research can help doctors make the right diagnosis and create a final treatment plan involving periodontal therapy with endodontic treatment using CGF. CGF has numerous benefits showing on bone which suggest that, under some circumstances, a single CGF treatment might be just as beneficial therapeutically as a mixture of grafting materials in endo-perio lesions. In the future, this would encourage the use of CGF alone rather than grafting materials for effective reduction in PPD and radiographic findings such defect depth and increase in bone volume. Furthermore, it was discovered that CBCT was a better option than invasive histologic evaluation.

## Ethics approval and consent participate:

The Ethics Committee of the Government Dental College and Hospital approved the project.

## Consent for publication:

Yes approved

## Funding:

There are none

## Author Contribution:

Sai Krishna Bingi - Contributed to conception, drafted and critically revised the manuscript, Give the final approval, agrees to be accountable for all aspects of work. K Sitamahalakshmi - Contributed to conception, design, data acquisition and interpretation, drafted and critically revised the manuscript, Give the final approval, agrees to be accountable for all aspects of work. Jaya Nagendra Krishna - Contributed to conception, design, drafted and critically revised the manuscript, Give the final approval, agrees to be accountable for all aspects of work. Shaik Imran - Contributed to conception, design, drafted and critically revised the manuscript, Give the final approval, agrees to be accountable for all aspects of work. Govardhani Krishnakumar - Contributed to conception, design, drafted and critically revised the manuscript, Give the final approval, agrees to be accountable for all aspects of work.

## Figures and Tables

**Table 1:** Plaque index

**SCORE 0**	**No plaque in the gingival area**
SCORE 1	A film of plaque adhering to free gingival margin and adjacent area of the tooth. The plaque may be recognized only by running a probe across the tooth surface.
SCORE 2	Moderate accumulations of soft deposits within the gingival pocket and on the gingival margin and / or the adjacent tooth surface that can be seen by the naked eye
SCORE 3	Abundance of soft matter within the gingival pocket and/or on the gingival margin and adjacent tooth surface.

**Table 2:** Gingival Index

**Mild gingival inflammation, slight changes in color, slight edema, no bleeding on probing**	**Mild gingivitis**
Moderate inflammation, redness, edema, bleeding on probing.	Moderate gingivitis
Sever inflammation, marked redness and edema, ulceration, tendency to spontaneous bleeding.	Severe gingivits

**Table 3:** plaque index, gingival index and periodontal pocket depth values from baseline to 9 months

**Parameter**	**Time**	**No of patients**	**Mean**
	Baseline	2	1.9
	9 months	2	0.9
**Plaque index(PI)**	**Baseline**	**2**	**1.9**
	9 months	2	0.9
	Baseline	2	2
	9 months	2	1
**Gingival index(GI)**	**Baseline**	**2**	**2**
	9 months	2	1
	Baseline	2	8.0mm
	9 months	2	6.0mm
**Periodontal pocket depth(PPD)**	**Baseline**	**2**	**8.0mm**
	9 months	2	4.0mm

**Table 4:** bone volume from base line to 9 months

**Parameter**	**Time**	**Mean**
Bone volume in cubic centimeters(cc)	Baseline	1.11
	9 Months	1.124
